# Expression and purification of the HTLV-1 transforming protein Tax

**DOI:** 10.1186/1742-4690-8-S1-A145

**Published:** 2011-06-06

**Authors:** Whitney Evans, Patrick Carriere, Morgan Weber, Lynez Preyan, Boguslawa Korona, Robert C Blake II, Vicki Traina-Dorge, Maureen Shuh

**Affiliations:** 1 Department of Psychology, Loyola University of New Orleans, New Orleans, LA, 70118, USA; 2 Division of Basic Pharmaceutical Sciences, College of Pharmacy, Xavier University of Louisiana, New Orleans, LA, 70125, USA; 3 Division of Microbiology, Tulane National Primate Research Center, Covington, LA, 70433, USA

## 

Human T cell lymphotropic virus, type 1 (HTLV-1), is the causative agent of adult T cell leukemia (ATL), a debilitating disease with a poor prognosis and no cure. HTLV-1 is a retrovirus which contains a 9 kb ssRNA genome, encoding the classic retroviral genes gag, pol, and env. HTLV-1 also encodes several regulatory genes, including tax. Tax is a 353 amino-acid, 40 kiloDalton (kDa) protein, located predominantly in the nucleus although a small percentage is isolated in the cytoplasm. Tax functions to regulate viral gene expression by interacting with the host transcription factor family, CREB/ATF. The Tax/CREB complex is formed on the 5’ LTR CRE site and activates viral gene transcription. In ATL cells (CD4+, CD8-), Tax also associates with two host transcription factors, NF-κB and SRF (serum response factor), resulting in the over-expression of host genes containing the NF-κB responsive element and the SRE (serum response element). The host genes, such as IL-2 and c-fos, are activated constitutively in the presence of Tax and result in cell proliferation of HTLV-1+ T cells, potentially leading to leukemia in the HTLV-1 patient. We believe that Tax activation of the SRF pathway serves as the initial transformation event of infected T cells. Thus, we are interested in the biochemical properties of Tax interactions with SRF, SRF/DNA, TCF (ternary complex factor, a binding partner with SRF), SRF/TCF, and SRF/TCF/DNA. Prior to conducting biochemical experiments, however, we need to express and purify Tax which has been difficult to accomplish in the HTLV-1 field and has impeded any progress toward studying the biophysical properties, including structure determination, of Tax. We present data showing our initial protein purification techniques that result in the isolation of a 99% pure, untagged, full-length Tax as well as the expression and purification of full-length, untagged human SRF.

